# Ground motion prediction equations as a proxy for medium properties variation due to geothermal resources exploitation

**DOI:** 10.1038/s41598-022-16815-x

**Published:** 2022-07-25

**Authors:** Vincenzo Convertito, Raffaella De Matteis, Ortensia Amoroso, Paolo Capuano

**Affiliations:** 1 Istituto Nazionale di Geofisica e Vulcanologia, Osservatorio Vesuviano, Via Diocleziano 328, 80124 Napoli, Italy; 2 Dipartimento di Scienze e Tecnologie, Università Degli Studi del Sannio, Via dei Mulini, 82100 Benevento, Italy; 3 Dipartimento di Fisica “E. R. Caianiello”, Università di Salerno, 84084 Fisciano (SA), Italy

**Keywords:** Solid Earth sciences, Seismology

## Abstract

Sub surface operations for energy production such as gas storage, fluid injection or hydraulic fracking modify the physical properties of the crust, in particular seismic velocity and anelastic attenuation. Continuously measuring these properties may be crucial to monitor the status of the reservoir. Here we propose a not usual use of the empirical ground-motion prediction equations (GMPEs) to monitor large-scale medium properties variations in a reservoir during fluid injection experiments. In practice, peak-ground velocities recorded during field operations are used to update the coefficients of a reference GMPE whose variation can be physically interpreted in terms of anelastic attenuation and seismic velocity. We apply the technique to earthquakes recorded at The Geysers geothermal field in Southern California and events occurred in the St. Gallen (Switzerland) geothermal field. Our results suggest that the GMPEs can be effectively used as a proxy for some reservoir properties variation by using induced earthquakes recorded at relatively dense networks.

## Introduction

The exploitation of new energy resources involving the crustal structure of the Earth naturally carries the risk of significantly perturbing the current stress field by leading to induced/triggered seismicity. When dealing with fluids, as in the case of geothermal fields, pore fluid pressure diffusion represents the main triggering mechanism. Indeed, for example, it can reduce the frictional resistance to sliding of the fault triggering an earthquake.

Field operations are designed to modify the physical properties of the rocks in order to increase the efficiency of the reservoir. This is the case, for example, of the Enhanced Geothermal Systems (EGSs) where an increase in the permeability is sought to make the exploitation more efficient. However, in addition to the permeability, it is reasonable to expect that other physical properties such as seismic velocity and anelastic attenuation can change, in particular when a large volume of fluids is involved in the field operations^1,2^.

Conventional techniques such as 4D seismic velocity, anelastic attenuation or seismic noise tomography, can be used for monitoring and interpreting medium properties variation (e.g.^3,4^). It has been shown that *V*_*P*_*/V*_*S*_-ratio obtained from seismic tomography is positively correlated to temporal changes in reservoir saturation and can thus be used to estimate and predict saturation changes in the reservoir^5^. However, these techniques are demanding in terms of data analysis and are time consuming (e.g.^5^).

In a recent paper, Convertito et al.^2^, through a synthetic test, have shown that the analysis and the modelling of the attenuation of the peak ground velocities, by using ground-motion prediction equations (GMPEs), can be used for monitoring the variations of the medium properties (see Supplementary Materials for details). In particular, the authors focused on the quality factor *Q*, which is sensitive to distinct physical parameters such as temperature, pore fluid pressure, degree of fracturing and state of stress/strain (e.g.^6,7^). The idea was to test if the coefficient generally adopted in the GMPE to model the effect of the anelastic attenuation (e.g.^8^) changes during the distinct stages of the project.

In the present paper we apply the approach originally tested on synthetic data^2^, on two real datasets. In particular, we analyse data recorded during fluid injection at The Geysers (California) geothermal field and at St. Gallen (Switzerland) deep geothermal field.

**Figure 1:**
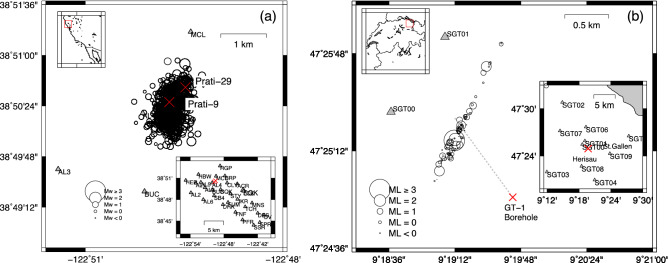
Geographic distribution of the analysed seismicity. (**a**) Epicentral distribution of the earthquakes and seismic network configuration for The Geysers, California, geothermal field. The two red crosses indicate the location of the Prati-9 and Prati-29 injection wells. (**b**) Epicentral distribution of the earthquakes and seismic network configuration for the St. Gallen, Switzerland, geothermal field. The location of the GT-1 well is indicated with a red cross. The dashed line represents the surface projection of the well trajectory.

**Figure 2:**
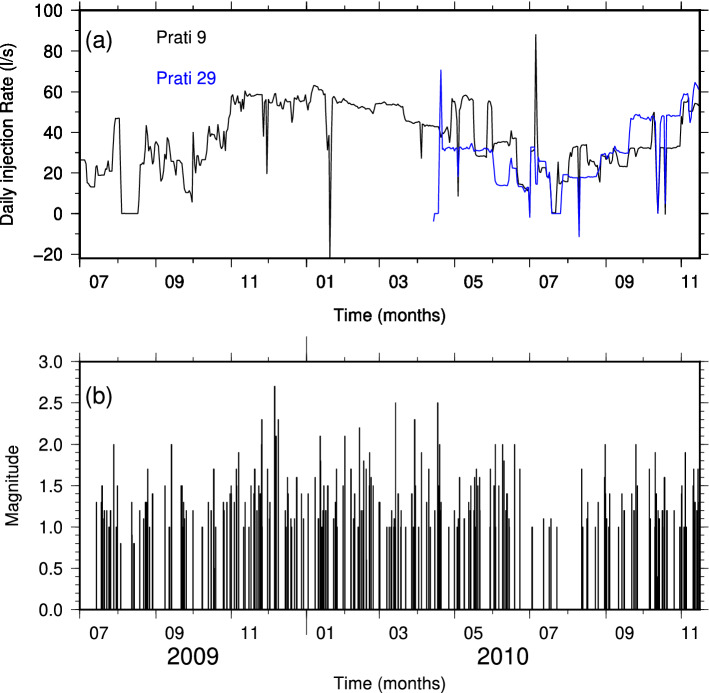
Injection rate and seismicity evolution for the Geysers. (**a**) Daily injection rate at Prati-9 and Prati-29 injection wells. (**b**) Temporal distribution of the seismicity recorded at The Geysers geothermal field in the period July 2009 November 2010.

The Geysers is a vapour-dominated geothermal field constantly operating in California since 1960s (e.g.^9,10^). In the present study, we focus our analysis on the earthquakes localized at a distance of about 1 km from the Prati 9 and Prati 29 wells in the period July 2009 through November 2010 (Fig. 1a). The Prati-9 well is used as a demonstration site for an Enhanced Geothermal System and the injection was continuous during the period of interest for the present study. The injection into Prati-29 initiated in April 2010 and was carried on until June 2013^11,12^ (Fig. 2a). The time evolution of the recorded seismicity is shown in Fig. 2b.

Concerning St. Gallen geothermal field, earthquakes were recorded by the network managed by Swiss Seismological Service^13,14^ originally consisting of one short period borehole sensor at 205 m depth and five additional broadband surface stations operated within a 12 km radius around the borehole. Seven additional short period surface stations were added in July 2013^15^. Overall, the data analysed in the present study, were recorded by 10 stations (Fig. 1b) (See “Methods” section for details).

**Figure 3:**
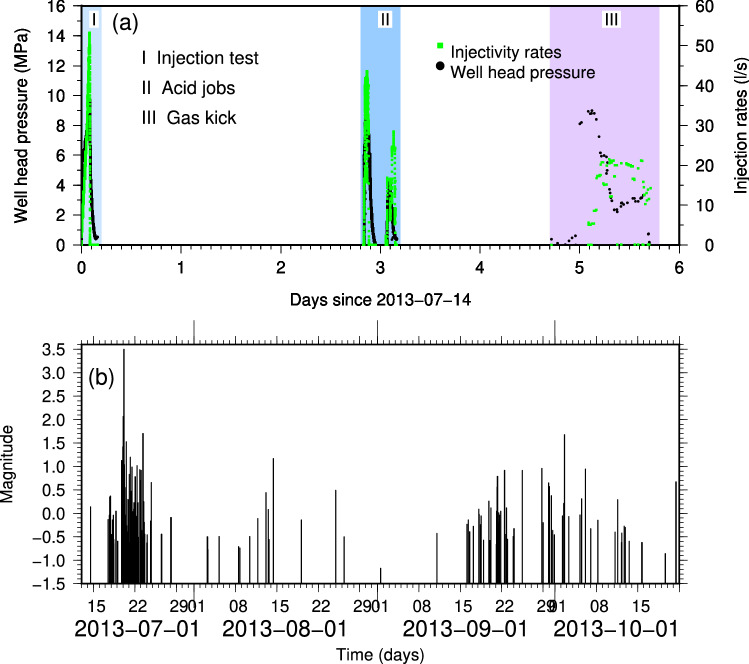
Injection rate and seismicity evolution for St. Gallen. (**a**) Well head pressure (black dots) and injection rates (green dots) during the different phases of the project as reported by Moeck et al.^16^. (**b**) Temporal distribution of the seismicity recorded at St. Gallen geothermal field.

The target of the project was a carbonate aquifer at a depth of ≈ 4 km to produce electricity and heating. As reported by Moeck et al.^16^ and Zbinden et al.^17,18^ the project started with a stimulation phase on 14th July 2013 during which water was injected at a rate of 53 l/s for a total amount of 175 m^3^ in 2 h. During the stimulation phase, only microseismicity with magnitude lower than 0.2, was induced. From 14 July through 19 July acid stimulations were performed involving about 290 m^3^ of fluids, which broke seal to gas reservoir and caused a gas kick (95% CH4). Afterwards, well control operations were done by injecting ~ 700 m^3^ of water and heavier liquids, which probably induced the largest event in the sequence with M_L_ 3.5 (Fig. 3a). Well control operations ended on 24 July. Despite this adverse consequence, in August 2013 a decision was made to continue the field operations with a high feeling of solidarity from the public. On September 2013 fishing operations were done together with a cleaning of the well^17^. The whole recorded seismicity is shown as function of time in Fig. 3b.

## Results

**Figure 4:**
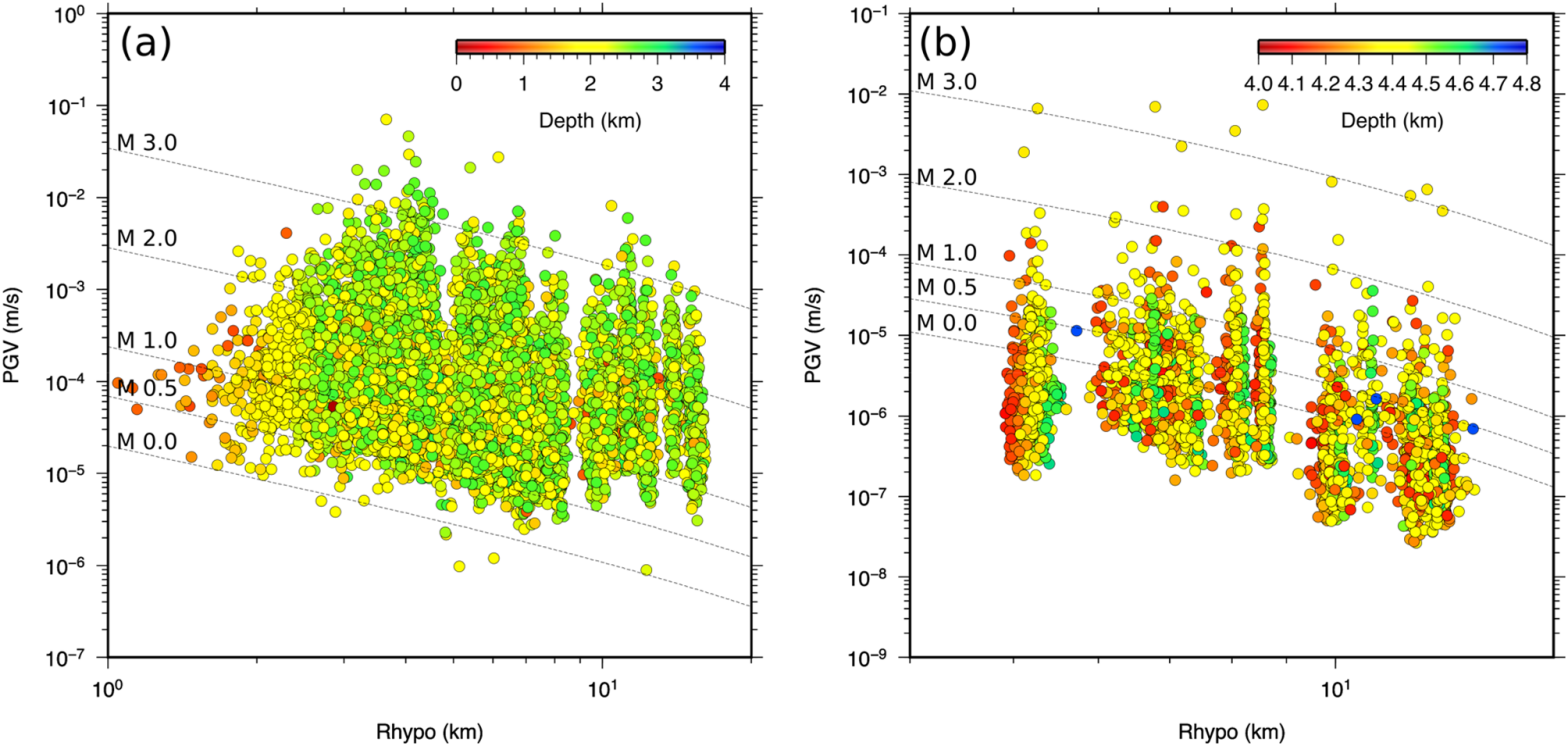
Peak-ground velocity distribution. (**a**) PGVs distribution as function of the hypocentral distance for The Geysers geothermal field. The values are colour coded according to the depth of the earthquake. The dashed lines represent the inferred reference GMPE for four magnitude values. (**b**) Same as panel (**a**) but for St. Gallen geothermal field.

As for The Geysers geothermal field, we first inferred a reference GMPE model using the PGVs plotted as function of the hypocentral distance in Fig. 4a (see “Methods” section). Next, we analysed peak-ground velocities (PGVs) by selecting earthquakes in consecutive time windows containing at least 15 events, which are used to update the coefficients of the GMPE. We note that the synthetic tests used to verify the proposed methodology^2^ have shown that in order to obtain stable results some of the coefficients of the GMPE, in particular those related to the geometrical spreading and to the site effect, should not be re-estimated when new data are collected. Thus, in the present analysis we consider only the coefficients *a* and *d* (see Eq. (1) in “Methods” section), being *d* related to the anelastic attenuation. In order to corroborate the results, we performed a comparison with independent estimates of *Q* inferred from source spectral analysis (see “Methods” section).﻿ Besides, for the analysed events, using the modified Wadati diagram approach (see “Methods” section), we also computed the *V*_*P*_*/V*_*S*_-ratio, which from the results of seismic tomography is correlated to temporal changes in reservoir saturation^1^. Due to the extent of the available catalogue, we computed the *V*_*P*_*/V*_*S*_-ratios by selecting earthquakes in consecutive 1-month long time windows. We note that while the d coefficient of the GMPE and *V*_*P*_*/V*_*S*_-ratio are computed by collecting events in each time window, *Q* is estimated for each single earthquake.

**Figure 5:**
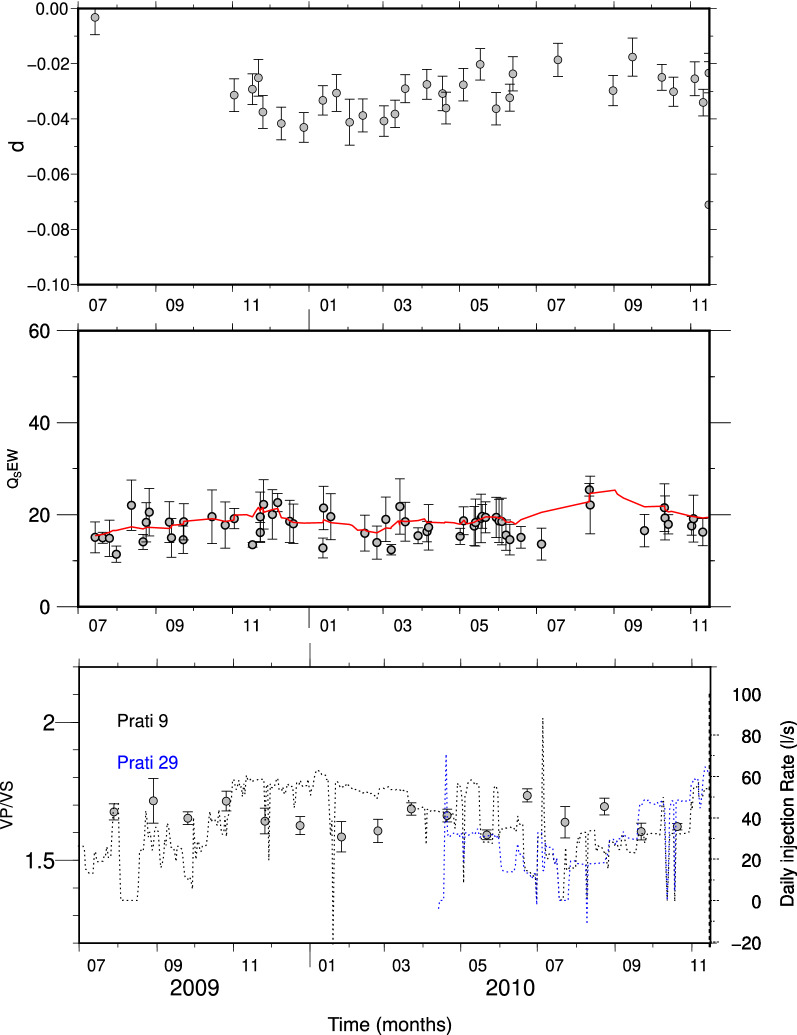
Time evolution of the inferred parameters. Upper panel: the *d* coefficient and related uncertainty obtained for The Geysers geothermal field. Central panel: *Q*_*S*_ quality factor estimated from the spectral analysis for each single earthquake. The red line is obtained by smoothing the single points. Lower panel: *V*_*P*_*/V*_*S*_-ratios and associated uncertainties obtained from the Wadati modified diagram. In the lower panel, the dashed line indicates the daily injection rate at Prati 9 well (black dashed line) and at Prati 29 well (blue dashed line).

**Figure 6:**
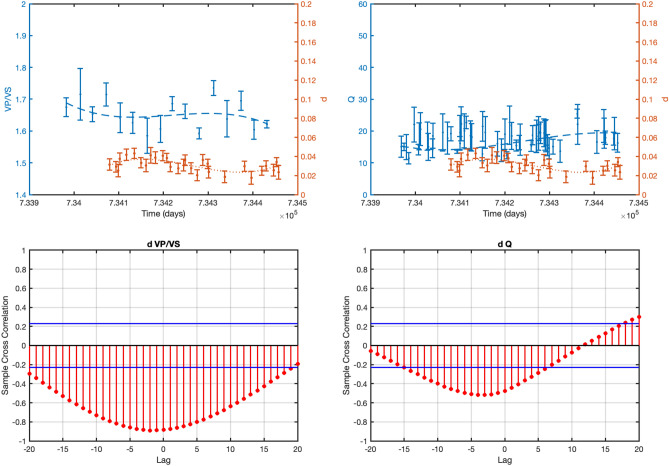
Cross correlation analysis for The Geysers geothermal field. The upper panels depict the data and the weighted polynomial fit for *d* (in absolute value), *V*_*P*_*/V*_*S*_ and *Q*_*S*_. The lower panels show the sample cross-correlation results for *d* and *V*_*P*_*/V*_*S*_ (left panel) and *d* and *Q*_*S*_ (right panel) for different time-lags (time-lag = 5 days). The horizontal lines represent the 2-sigma confidence bounds.

For all the three parameters, namely, *d*, *Q*_*S*_ and *V*_*P*_*/V*_*S*_-ratio, we discarded the values with a relative error ($\frac{\delta x}{\left|x\right|}$ being $\delta x$ the standard error of each parameter) larger than 0.4. The results are plotted in Fig. 5 and suggest a correlation between the three parameters. In order to have a quantitative estimation, we computed the sample cross-correlation. However, since the three time-series are sampled at different and not constant rates, we first fitted (weighted fit) them by using polynomial functions and then computed the cross-correlation. The results are shown in Fig. 6 and demonstrate that a significant correlation does exist between *d* and *V*_*P*_*/Vs,* and *d* and *Q*_*S*_ within 2-standard-error confidence bounds. For completeness we report the inferred *a*-values in Fig. S2 in the Supplementary Materials.

Besides, it can be noted how the same parameters are clearly anticorrelated with the injection rate (Fig. 5). The higher the injection rate the lower are both the anelastic attenuation and the *V*_*P*_*/V*_*S*_-ratio suggesting a relevant effect of fluids on rock properties. As a final observation, we note that the *Q*_*S*_ values estimated by using the spectral analysis agree with the tomography results, which provide *Q*_*S*_ values ranging between 15 and 50 at the base of the Prati 9 well^6^.

**Figure 7:**
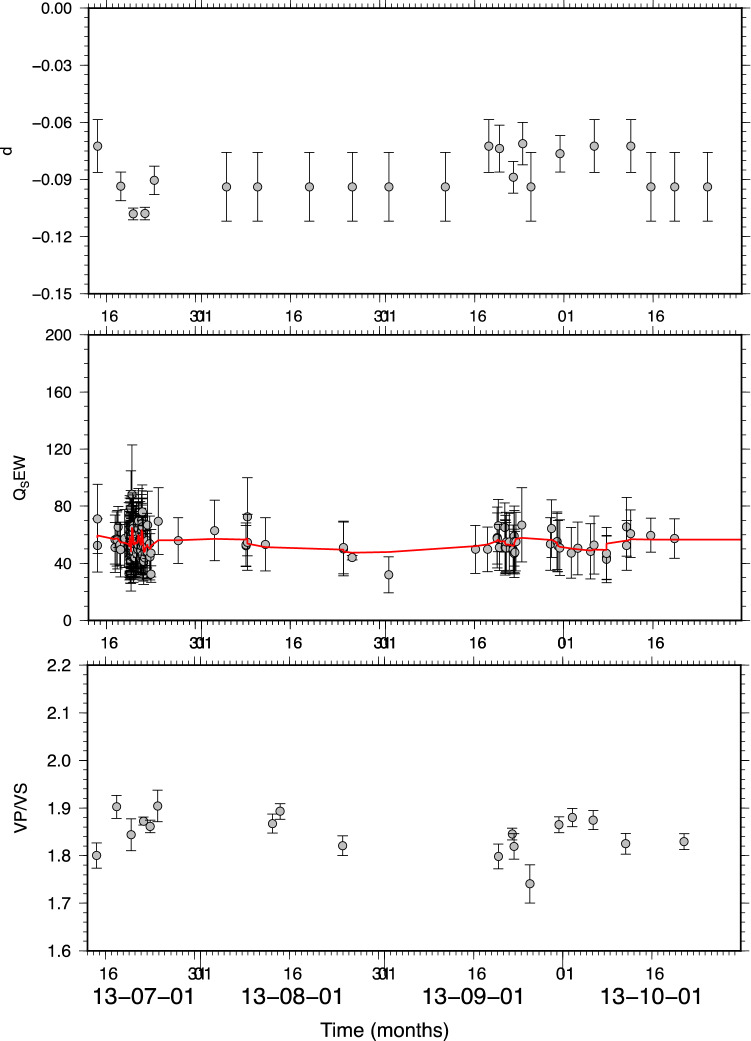
Same as Fig. 5 but for St. Gallen geothermal field for the whole catalogue.

**Figure 8:**
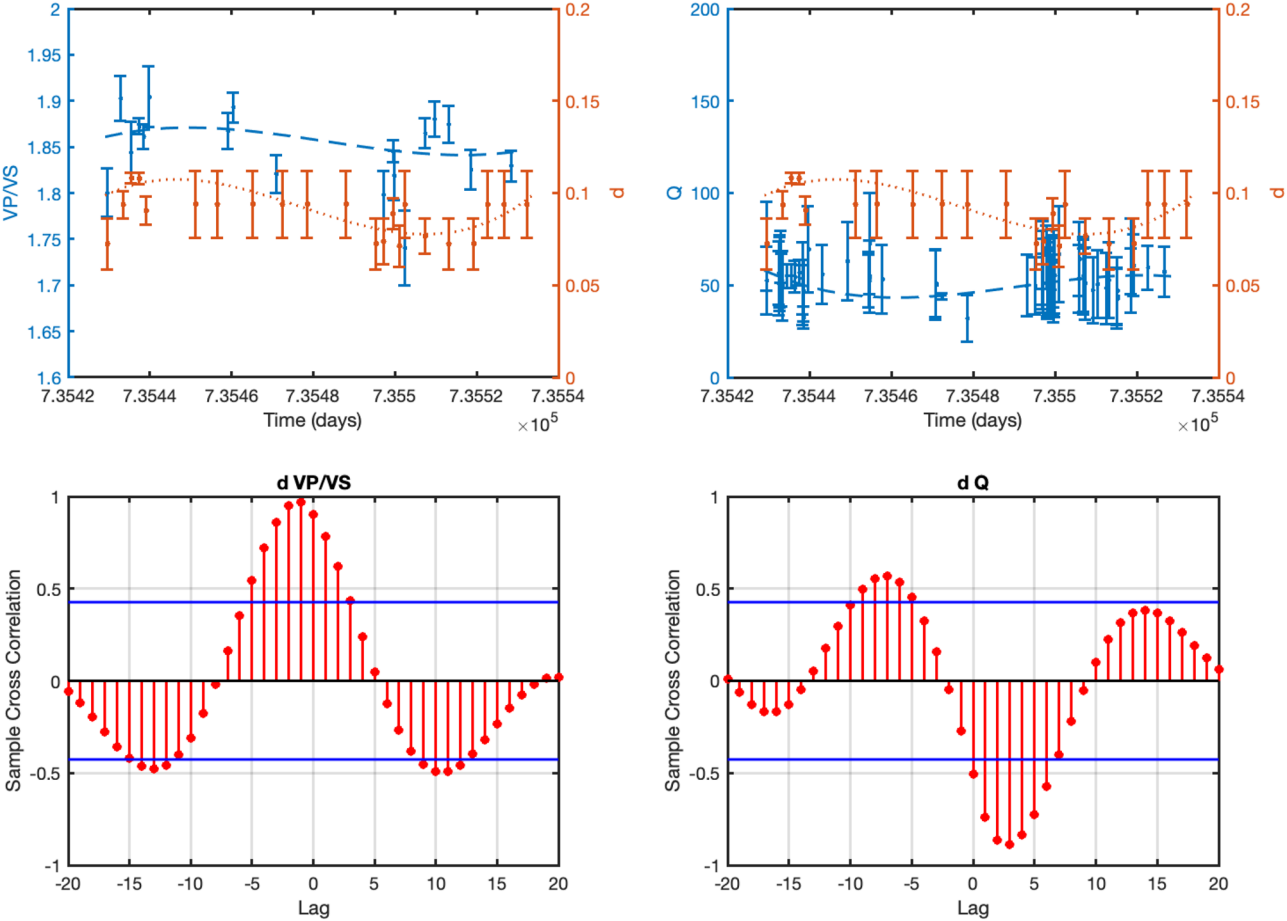
Same as Fig. 6 but for St. Gallen geothermal field (time-lag = 5 days).

As for the St. Gallen geothermal field, we first inferred a reference GMPE model using the PGVs plotted in Fig. 4b (see “Methods” section). We considered the whole catalogue and selected earthquakes in contiguous 2 days wide time windows. Similar to the previous analysis *Q*_*S*_ is estimated for each single earthquake. The results of the analysis are reported in Fig. 7 (the inferred a-values are shown in Figure S2 in the Supplementary Materials) while Fig. 8 shows the cross-correlation analysis performed by following the same approach used for The Geysers. Similarly, for St. Gallen the correlation between *d* and *V*_*P*_*/Vs*, and *d* and *Q*_*S*_ is significant within 2-standard-error confidence bounds.

Furthermore, since we have information about the field operations for the initial phase of the project, namely, covering only about 6 days since the beginning of the operations on 14^th^ July 2013, we repeated the analysis for this specific period. Because most of the seismicity occurs in this time-window, we computed *d*, *V*_*P*_*/Vs* by selecting earthquakes in 2 h contiguous time windows.

**Figure 9:**
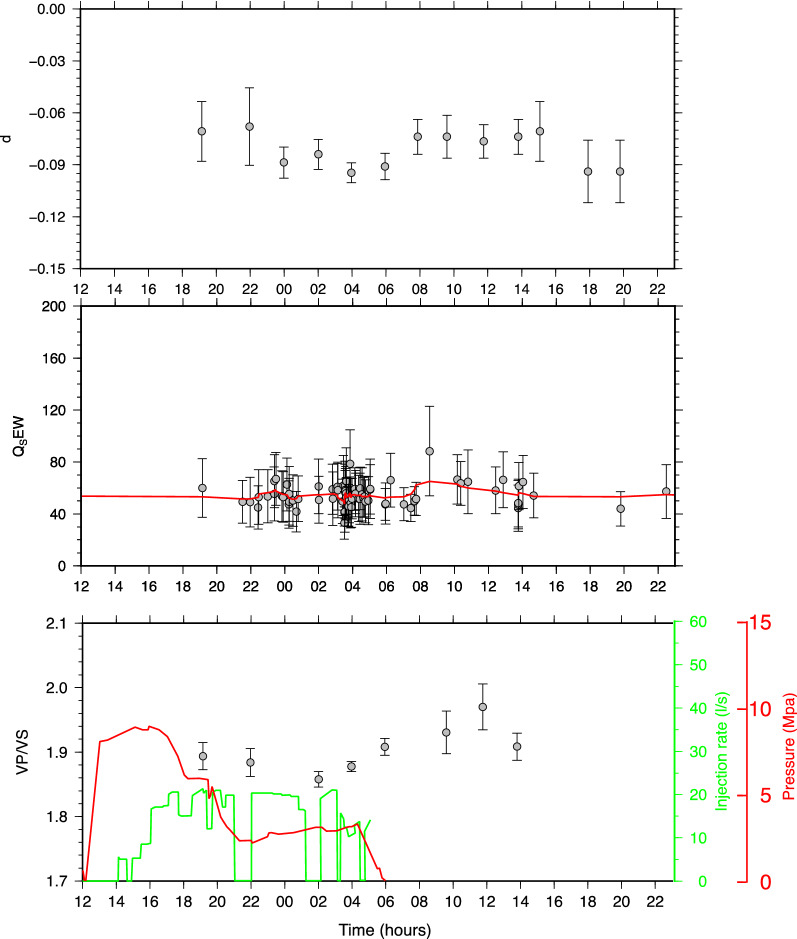
Same as Fig. 5 but for St. Gallen geothermal field relative to the time period 2013/07/19 12:00:00 up to 2013/07/20 23:00:00.

**Figure 10:**
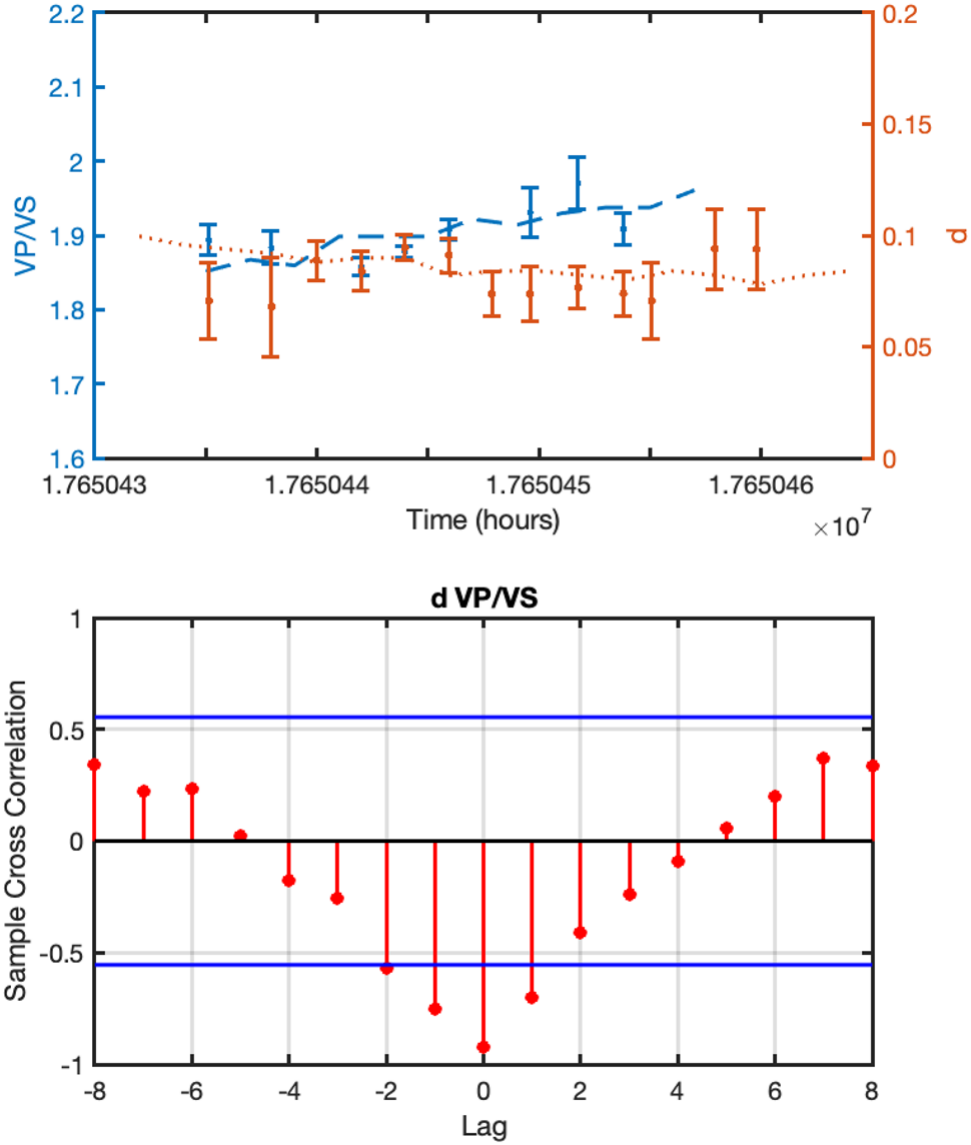
Same as Fig. 6 but for St. Gallen geothermal field and relative to the time period 2013/07/19 12:00:00 up to 2013/07/20 23:00:00 (time-lag = 2 h).

The results are shown in Fig. 9 and indicate that the *d* coefficient varies following the wellhead pressure profile and seems to be correlated to the *V*_*P*_*/V*_*S*_-ratio. This is confirmed by the cross-correlation analysis shown in Fig. 10. As for *Q*_*S*_ the results show that its variations—between 32 and 88—are contained in the error bar except for a few points not showing any significant trend that can be correlated with *d*.

## Discussion

We have proposed a not usual way of using the ground motion prediction equations (GMPEs) that have been originally developed to provide a tool for estimating peak-ground motion parameters (e.g., Peak Ground Acceleration, Peak Ground Velocity, etc.…). In particular, we used the GMPEs to monitor induced seismicity data and their effect on the crustal medium embedding the reservoir, during field operations aimed at producing geothermal energy. In fact, reservoir monitoring is generally performed by analyzing the variation of the rock properties in terms of elastic parameters such as the seismic velocity (e.g.^7^). Fluids saturation, pore fluid pressure variations, for example, can affect both P- and S-wave velocity (e.g.^19^). However, laboratory measurements (e.g.^20,21^) and data analysis recorded during field operations^6,22^ suggest that the same phenomena can also affect the anelastic attenuation of the rocks since anelastic attenuation is generally assumed to be due to inter-crack motion or fluid flow between pores depending on the rock type (e.g.^19^). In particular, for sedimentary rocks attenuation depends on both fluid saturation and differential pressure. For crystalline rocks the temperature has a prominent effect together with grain boundary sliding and movement of dislocation^6^. Moreover, it is suggested that *Q* is more sensitive to rock properties related to pores, cracks, fractures and fluids compared to the compressional and shear wave velocities^7^.

While 4D seismic tomography based on seismic phases travel-times can be implemented to monitor seismic velocity changes, measuring anelastic attenuation changes requires the analysis of waveform features that are more onerous to obtain. This is the case, for example of the first P rise-time or the total pulse P-wave (e.g.^23^), the analysis of the coda-waves (e.g.^24^), and the spectral analysis (e.g.^25–28^). Synthetic tests performed by Convertito et al.^2^ have shown that the GMPEs are sensitive to the variation of the status of the reservoir in terms of anelastic attenuation and seismic velocity. To complement the synthetic tests here we report the results for two geothermal fields, that is, the Geysers in California and the St. Gallen geothermal field in Switzerland.

For the two study areas, the results indicate that the *d* coefficient of the GMPE is sensitive to the field operations. This is corroborated by the correlation of *d* with the *V*_*P*_*/V*_*S*_-ratio and *Q*_*S*_ parameters that have been proved to be sensitive to the perturbations of the crustal medium.

As for the Geysers geothermal field, it has been theorized that when injected water contacts hot reservoir rock, heat is drawn from the reservoir rock until the water vaporizes^1^. The resulting cooling and contraction of the rock generates tensile (mode I) cracks and subsequent microseismicity^1,6^. The cold water in hot material in addition to causing rock fracturing, produces a partial saturation of the rocks—at least before the water turns to steam—which could account for the observed low *Q*_*S*_ values^6^. However, our results indicate that, while the seismicity is correlated with the injection rate, *V*_*P*_*/V*_*S*_-ratio is clearly anticorrelated with the injection rate (Fig. 5). The decrease in the *V*_*P*_*/V*_*S*_-ratio with the increasing of the injection rate suggests that, whatever the mechanism, it produces a decrease in *V*_*P*_ that is higher than the decrease of the *V*_*S*_ likely caused by the presence of fluids with a high percentage of steam.

As for the St. Gallen geothermal field, our results, relative to the period for which detailed information about filed operations are available, suggest that the *d* coefficient and the *V*_*P*_*/V*_*S*_-ratio are correlated with the wellhead pressure values (Fig. 6).

This result must be interpreted by taking into account the physical mechanism by which the earthquakes are induced. At St. Gallen geothermal field, earthquakes have been likely originated from the effect of over pressurized gas^18^ due to an unexpected event of gas kick, occurred on July 19^th^ at about noon. The gas kick altered the hydraulic conditions by favoring the seismic sequence migration in a specific direction^15^. Thus, it can be reasonably assumed that the high concentration of gas could be the origin of the low *V*_*P*_*/V*_*S*_-ratio observed after the gas kick (Fig. 6) up until 6 am on 20 July. Afterwards, the parameters start to increase again suggesting the prevalence of the liquid phase with respect to the previous gas phase.

To conclude, our results suggest that the *d* coefficient of the GMPE is an indicator of the medium properties changes having observed that, for the two geothermal areas, it is correlated with parameters (i.e., *V*_*P*_*/V*_*S*_-ratio and *Q*_*S*_), that are physically interpretable in terms of crustal medium properties. Thus, if future studies should demonstrate that critical values of *V*_*P*_*/V*_*S*_-ratio and *Q*_*S*_ parameters can be used by the operators to take actions for mitigating induced seismicity—as for example by reducing injection rate—then also the *d*-coefficient could be used as an additional parameter to be monitored.

The proposed technique has the advantage that it requires only the location of the events and the measure of the PGVs that, compared to other measures, are more readily measured after the earthquake occurrence.

## Methods

### Reference GMPE

As for The Geysers geothermal field, we used waveforms recorded at 29 seismic stations of the network managed by the Lawrence Berkeley National Laboratory (LBNL) Geysers/Calpine (BG). The BG stations were equipped with I/O Sensor SM-6 geophones with a natural frequency of 14 Hz. In the fall of 2009, these instruments were replaced by Oyo GS-11D 4.5 Hz sensors. In the selected period 745 earthquakes were recorded with moment magnitude ranging between 0.3 and 2.7. The available PGVs are shown in Fig. 4a. We note that the available data did not allow to include the station/site effect in the GMPE thus the adopted model is the one reported in the following:

1 $$\log PGV = a + bM + eM^{2} + c\log R + dR$$

where *M* is the magnitude, *R* is the hypocentral distance in km, *c* is the coefficient that accounts for the geometrical spreading, and *d* is the coefficient that accounts for the anelastic attenuation (e.g.^29^). We have verified through the Akaike criterion^30^ that a model with the additional square dependence on the magnitude performs better than the model with only the linear dependence. The coefficients together with their uncertainty and the total standard error are listed in Table 1. In the last column we also report the Variance Inflation Factor (VIF), which allows to test the multicollinearity in a set of model parameters. As can be noted, the VIF for the *d*-coefficient is less than 1 indicating no correlation between *d* and any other inferred coefficient^31^.

**Table 1:** Coefficients, related uncertainty and Variance Inflation Factor (VIF) of the GMPE reported in Eq. (1) for The Geysers.

Parameter		VIF
*a*	− 4.247 ± 0.051	23.7915
*b*	0.549 ± 0.063	0.9716
*e*	0.177 ± 0.020	26.6670
*c*	− 1.096 ± 0.064	15.2874
*d*	− 0.017 ± 0.004	0.2807
*s*	**–**	
$\sigma$	0.395	

In order to infer the reference model for the St. Gallen geothermal field we adopted the two-step approach proposed by Emolo et al.^32^ and Sharma et al.^33^. We analysed the waveforms of 346 events with magnitude ranging between − 1.2 and 3.5 and depths ranging between 4.4 and 4.7 km (Fig. 1a) reported by Diehl et al.^15^, and recorded by 10 stations (Fig. 1b). The 9 surface 3-component stations are equipped with Nanometrics Trillium Compact 120 s (SGT01-SGT05) and short period 1 s (SGT06–SGT09). The bore-hole station SGT00 is equipped with an OYO Geospace HS-1LT sensor (2.0–28 Hz).

The available peak-ground velocities (PGVs) are shown in Fig. 4b. In practice, in the first step we selected the model reported in Eq. (1) and did not include the borehole station. In the second step, in order to include in the Eq. (1) site/station effects, we compute the residuals distribution at each station including the borehole station. We then use the modal value as site/station effect that can be thus included in Eq. (1). The new equation is given by:

2 $$\log PGV = a + bM + eM^{2} + c\log R + dR + st$$

The inferred coefficients together with the standard error and the total standard error are listed in Table 2. The coefficients are obtained by using the Levenberg–Marquardt least squares algorithm^34^ for curve fitting. In the last column we also report the Variance Inflation Factors, which for the *d*-coefficient is less than 5 indicating a slight-to-moderate correlation, which however is not severe enough to require attention^31^. The *t*-values for all the stations are reported in Table 3.

**Table 2:** Coefficients and related uncertainty, and Variance Inflation Factor (VIF) of the GMPE reported in Eq. (2) for St. Gallen.

Parameter		VIF
*a*	− 3.833 ± 0.189	766
*b*	0.793 ± 0.011	1.4
*e*	0.069 ± 0.007	1.4
*c*	− 1.206 ± 0.388	2757
*d*	− 0.100 ± 0.019	2.1
*s*	0.687 ± 0.021	698
$\sigma$	0.298

**Table 3:** Station/site correction coefficients to be used in Eq. (2).

Station code	t-value
SGT00	− 0.75
SGT01	− 0.39
SGT02	− 0.33
SGT03	0.1
SGT04	− 0.48
SGT05	− 0.28
SGT06	0.05
SGT07	0.32
SGT08	0
SGT09	0.16

### Estimation of the quality factor *Q* from spectral analysis

We adopted the approach proposed in Zollo et al.^27^ to infer the *Q* parameters from the analysis of the displacement spectra. In particular, we assume the following spectral shape:

3 $$S\left(f\right)=\frac{{\Omega }_{0}}{1+{\left(\frac{f}{{f}_{c}}\right)}^{\gamma }}{e}^{-\frac{\pi Tf}{Q}}$$

where *T* is the travel time of the selected seismic phase (S-wave in the present study), *γ* defines the spectral decay, *f*_*c*_ is the corner frequency and *Q* is the quality factor, which controls the anelastic attenuation. The ${\Omega }_{0}$ parameter represents the spectral level at low frequency and is used to compute the seismic moment. For small magnitude events we can reasonably assume that for *f* <  < *f*_*c*_ Eq. (3) can be written as:

4 $$lnS\left(f\right)=ln{\Omega }_{0}-\frac{\pi Tf}{Q}$$

Thus, using a lin-log representation of the spectrum, the quality factor *Q* can be obtained from the slope of the linear fit.

### The *V*_*P*_/*V*_*S*_ ratio estimation from the Wadati diagram

We implemented the modified Wadati diagram^35^, which provides an estimate of the average *V*_*P*_/*Vs* ratio. The diagram is obtained by considering for each event, and for each pair of station (*i*, *j*), the difference between *P*-phase (*T*_*Pi*_ − *T*_*Pj*_, *x*-axis) and *S*-phase (*T*_*Si*_ − *T*_*Sj*_, *y*-axis) arrival times. The advantage of the modified Wadati diagram is that it does not depend on the earthquake origin time. It can be shown that:

5 $${T}_{{S}_{i}}-{T}_{{S}_{j}}=\frac{{V}_{P}}{{V}_{S}}\left({T}_{{P}_{i}}-{T}_{{P}_{j}}\right)$$

Which indicates that from the slope of the line it is possible to estimate the *V*_*P*_*/V*_*S*_-ratio.

## Supplementary Information

Supplementary Information.

## Data Availability

Industrial data and waveforms analyzed in the present study for measuring the peak-ground velocities are available at IS EPOS (2017), Episode: THE GEYSERS Prati 9 and Prati 29 cluster, https://tcs.ah-epos.eu/#episode:THE_GEYSERS_Prati_9_and_Prati_29_cluster, doi:10.25171/InstGeoph_PAS_ISEPOS-2017-011, and at IS EPOS (2018), Episode: ST. GALLEN, https://tcs.ah-epos.eu/#episode:ST_GALLEN, doi:10.25171/InstGeoph_PAS_ISEPOS-2018-007, last access 22 June 2022.

## Code availability

Computer codes developed for the present study are available upon request.
