# Supplementary material for: Ground motion prediction equations as a proxy for medium properties variation due to geothermal resources exploitation
Source: Sci Rep. 2022 Jul 25;12:12632. doi: 10.1038/s41598-022-16815-x (PMC9314424; doi:10.1038/s41598-022-16815-x)
Supplement: Supplementary file 1 — Supplementary Information. [file 41598_2022_16815_MOESM1_ESM.docx]

**Supplementary material**

**Ground motion prediction equations as a proxy for medium properties variation due to geothermal resources exploitation**

**Authors**

Vincenzo Convertito^1^, Ortensia Amoroso^3^, Paolo Capuano^3^, and Raffaella De Matteis^2^

**Affiliations**

^1^Istituto Nazionale di Geofisica e Vulcanologia, Osservatorio Vesuviano, Via Diocleziano 328, 80124 Napoli, Italy (vincenzo.convertito@ingv.it),

^2^Dipartimento di Scienze e Tecnologie, Università degli Studi del Sannio, Via dei Mulini, 82100, Benevento, Italy (dematt@unisannio.it),

^3^Dipartimento di Fisica "E. R. Caianiello", Università di Salerno, 84084, Fisciano (SA), Italy (pcapuano@unisa.it).

^3^Dipartimento di Fisica "E. R. Caianiello", Università di Salerno, 84084, Fisciano (SA), Italy (oamoroso @unisa.it).

**Synthetic tests**

In this section we briefly report the results of the synthetic tests performed by Convertito et al.^1^ aimed to demonstrate the effectiveness of the technique proposed in the present manuscript.

In the synthetic test, the authors simulated the expected seismic activity (in terms of rate, focal mechanisms, stress drop and the b value of the Gutenberg-Richter) together with the expected changes in the crustal model through the quality factor *Q* possibly induced during a fluid injection experiment. They simulated full waveforms by using the AXITRA code^2^ and a triangle source-time function to represent the earthquake source. Peak-ground velocity (PGV) measured on the waveforms are simulated for both homogeneous and layered crustal models in which the *Q* values are changed. For example, for the layered model *Q* varies from 50 to 10 in first layer and from 500 to 100 in deeper part of the model at 7600 m.

The PGVs are used to update the coefficients of a reference GMPE in order to test whether the coefficients are able to capture the medium properties variation. In particular, the tests have suggested that the coefficients that are related to the geometrical attenuation and to the magnitude must be keep fixed, while the coefficients mainly related to stress-drop and to the anelastic attenuation can be inverted while new data are gathered during the field operations. Besides, the tests indicated that reliable results can be obtained when each event is inverted separately and that small magnitude earthquakes are more informative about the changes in the anelastic attenuation with respect to moderate events.

We report a figure modified from Convertito et al.^1^, which summarize the above discussion. In particular, the figure depicts the Results of the sensitivity test of the coefficients (*a*, *d*, and the total standard deviation) of the GMPE when data of each earthquake are separately inverted. The representation of the coefficients as function of the magnitude supports the conclusion about the above-mentioned difference between small and relatively larger earthquakes. The same figure clearly shows how the variation of *Q*, which decrease from the first crustal model (MOD1) to the third model (MOD3) is properly captured by the d-coefficient. In fact, the d-coefficient decreases as *Q* decreases.

**Supplementary References**

1. Convertito, V., De Matteis, R., Esposito, R. & Capuano, P. Using ground motion prediction equations to monitor variations in quality factor due to induced seismicity: a feasibility study. *Acta Geophys*. **68**, 723–735, <https://doi.org/10.1007/s11600-020-00441-0> (2020).
2. Cotton, F., and O. Coutant, Dynamic stress variations due to shear faults in a plane layered medium, *Geophys. J. Int.*, **128**, 676 – 688, (1997).

**Figure S1:** **Sensitivity test.** Sensitivity test of the coefficients of the GMPE when data of each earthquake are separately inverted. The coefficients are shown as function of the magnitude for the three distinct crustal model in which *Q* is gradually decreased (from MOD1 through MOD3) in the layers where most of the seismicity is located. The values of *Q* in each layer are also shown in the right panels. The horizontal lines represent the reference value of the indicated parameter while the black squares correspond to the mean value computed by binning the magnitude values.

**Figure S2: The a-coefficient.** Inferred a-coefficient as function of time for The Geysers geothermal field (upper panel) and for The St Gallen geothermal field (lower panel).
